# Influence of Static Magnetic Field Stimulation on the Accuracy of Tachystoscopically Presented Line Bisection

**DOI:** 10.3390/brainsci10121006

**Published:** 2020-12-18

**Authors:** Hikari Kirimoto, Tatsunori Watanabe, Nami Kubo, Shota Date, Toru Sunagawa, Tatsuya Mima, Katsuya Ogata, Hisato Nakazono, Shozo Tobimatsu, Antonio Oliviero

**Affiliations:** 1 Department of Sensorimotor Neuroscience, Graduate School of Biomedical and Health Sciences, Hiroshima University, Hiroshima 7348553, Japan; twatan@hiroshima-u.ac.jp (T.W.); m193054@hiroshima-u.ac.jp (N.K.); 2 Department of Analysis and Control of Upper Extremity Function, Graduate School of Biomedical and Health Sciences, Hiroshima University, Hiroshima 7348553, Japan; sdate@hiroshima-u.ac.jp (S.D.); torusuna@hiroshima-u.ac.jp (T.S.); 3 Graduate School of Core Ethics and Frontier Sciences, Ritsumeikan University, Kyoto 6038577, Japan; t-mima@fc.ritsumei.ac.jp; 4 Department of Speech and Hearing Sciences, Faculty of Health and Medical Sciences, International University of Health and Welfare, Fukuoka 8318501, Japan; ogata_k@iuhw.ac.jp; 5 Department of Occupational Therapy, Fukuoka International University of Health and Welfare, Fukuoka 8140001, Japan; nakazono@takagigakuen.ac.jp (H.N.); tobi@takagigakuen.ac.jp (S.T.); 6 FENNSI Group, Hospital Nacional de Paraple’jicos, SESCAM, 45071 Toledo, Spain; antonio.oliviero@hotmail.com

**Keywords:** transcranial static magnetic field stimulation, non-invasive brain stimulation, visuospatial cognition, unilateral spatial neglect

## Abstract

Transcranial static magnetic stimulation (tSMS) has been known to reduce human cortical excitability. Here, we investigated whether tSMS would modulate visuo-spatial cognition in healthy humans. Subjects performed a visuo-spatial task requiring judgements about the symmetry of pre-bisected lines. Visual stimuli consisted of symmetrically or asymmetrically transected lines, tachystoscopically presented for 150 ms on a computer monitor. Task performance was examined before, immediately after, and 10 min after tSMS/sham stimulation of 20 min over the posterior parietal cortex (PPC: P4 from the international 10–20 system) or superior temporal gyrus (STG: C6). Nine out of 16 subjects misjudged pre-bisected lines by consistently underestimating the length of the right-side segment (judging lines to be exactly pre-bisected when the transector was located to the left of the midpoint, or judging the left-side segment to be longer when the transector was located at the midpoint). In these subjects showing a leftward bias, tSMS over the right STG reduced the magnitude of the leftward bias. This did not occur with tSMS over the right PPC or sham stimulation. In the remaining right-biased subjects, no intervention effect was observed with any stimulation. Our findings indicate that application of tSMS over the right STG modulates visuo-spatial cognition in healthy adults.

## 1. Introduction

In a last decade, a number of studies have demonstrated that the excitability of cerebral cortices is suppressed by “transcranial static magnetic field stimulation (tSMS)” [1], in which the scalp is exposed to moderate-intensity (about 100 to 200 mT) static magnetic fields (SMFs) by a strong cylindrical neodymium, iron and boron (NdFeB) permanent magnet. Experimental evidence clearly indicates such suppressive effect in primate as well as non-primate animals [2,3]. With the growth of research activities, this novel method has recently been recognized as a member of inhibitory non-invasive brain stimulation (NIBS) techniques, such as low-frequency repetitive transcranial magnetic stimulation (rTMS) [4], continuous theta-burst stimulation [5], and cathodal transcranial direct current stimulation (tDCS) [6].

Given that the magnet used for tSMS is safer, cheaper, and easier to use as compared to the other conventional NIBS techniques, tSMS has the attractive potential to be used in clinical practice to alleviate symptoms of various central nervous system diseases. For instance, in stroke patients tSMS may be effective in restoring the sensorimotor function of paralyzed limbs by suppressing excessive inter-hemispheric inhibition from the intact hemisphere to the affected hemisphere via the corpus callosum [1,7,8,9,10,11,12,13,14]. Also, tSMS’s ability to reduce the amplitude of intra-epidermal electrical stimulation-evoked potentials [15] may lead to the development of chronic pain management. Furthermore, findings that tSMS over the supplementary motor area (SMA) can modulate resting-state functional magnetic resonance imaging (fMRI) activity [16] and the function of postural adjustments [17] indicate its potential use as a therapeutic technique to suppress excessive activity of the SMA, for example, in patients who have such neurological disorders as Parkinson’s disease and Gilles de la Tourette syndrome [18,19,20]. In addition, the suppression of epileptic seizures by tSMS observed in rats and monkeys [3] seems to be applicable even to humans.

One of the important functions that deserves attention but has not been addressed so far in terms of the effect of tSMS is visuo-spatial function. In particular, a failure to attend or act in one part of space after a stroke, called unilateral spatial neglect (USN), is of special interest, because it is associated with poor functional outcomes and length of stay in the hospital [21]. Anatomical brain regions responsible for this disorder have been highlighted in many previous studies such as the parietal cortex [22,23], superior temporal gyrus (STG) [24], and/or inferior frontal cortex [25,26] in the right hemisphere, and an increasing number of reports have shown that NIBS is effective in the treatment of USN in post-stoke patients [27,28,29,30,31,32]. Specifically, inhibitory rTMS over the intact hemisphere, especially over the right posterior parietal cortex (PPC), can reduce its pathologic hyperactivity occurring due to a deficit in transcallosal inhibition from the affected hemisphere [33]. The transient inhibition of the intact hemisphere is expected to decrease the interhemispheric imbalance and thus symptoms of USN. Even in healthy adults who commonly display a leftward bias (pseudoneglect), inhibitory rTMS over the right PPC was demonstrated to reduce the bias in symmetry judgements on pre-bisected lines [34]. Given the inhibitory characteristic of tSMS, as a first step towards clinical applications of this new tool in this area, we investigated whether tSMS on the right PPC or STG would have the same effect of reducing a bias in healthy adults in the present study. Accordingly, the study aim was to investigate the possibility of non-invasive modulation of visuo-spatial function by application of tSMS over the parietal or temporal cortex in healthy humans. If tSMS can modulate visuo-spatial bias in these individuals, it may become a novel, economical, convenient, and non-invasive method to alleviate symptoms of USN in post-stroke patients.

## 2. Materials and Methods

### 2.1. Subjects

Sixteen healthy subjects (10 males and 6 females, 21–39 years old) participated in this study. None were undergoing medical treatment for any condition. Based on administration of the Oldfield inventory [35], the handedness scores of all subjects ranged from 0.9 to 1.0 (strongly right-handed). Written informed consent was obtained from all participants before beginning the experiment, which was conducted according to principles of the Declaration of Helsinki. The experimental protocol was also approved by the Ethical Committee for Clinical Research of Hiroshima University (No. C-242).

### 2.2. Experimental Procedure

#### 2.2.1. Intervention Experiment

The Subject was seated comfortably in front of a computer monitor with a distance of 50 cm. The subject’s eye level was adjusted to be at the middle of the computer monitor that was centred on his/her sagittal midplane. The subject performed a line length judgement task requiring forced-choice decision about the symmetry of pre-bisected lines. Visual stimuli consisted of symmetrically or asymmetrically transected lines, which were tachystoscopically presented on the computer monitor. Task performance was examined before (pre), immediately after (post), and 10 min after (post 10) tSMS or sham stimulation of 20 min.

#### 2.2.2. Preliminary Experiment

Prior to the intervention experiment using tSMS/sham, 11 of 16 subjects (6 males and 5 females) performed the task with the stimulus presentation time of 50, 150, and 300 ms twice in a random order (6 sessions in total), to examine learning effects and performance reproducibility, and also to determine the appropriate stimulus presentation time. The stimulus presentation time of 150 ms, which showed the highest reproducibility, was used in the intervention experiment. This preliminary experiment was conducted more than a week before the intervention experiment.

### 2.3. Transcranial Static Magnetic Stimulation (tSMS)

For tSMS, we used a cylindrical NdFeB neodymium magnet (diameter, 50 mm; height, 30 mm) with a surface magnetic flux density of 534 mT, maximum energy density of 49 MGOe, and a strength of 862 N (88 kgf) nominal value (NeoMag, Ichikawa, Japan). We previously showed that this magnet generates a magnetic field that accesses most cortical regions (strength 110–90 mT at 2–3 cm from the surface of the magnet) and elicits biological effects [13]. For sham stimulation, we utilized a non-magnetic stainless-steel cylinder of the same size and weight. With the aid of an arm-type lightning stand (C-stand, Avenger, Cassola, Italy), the magnet or sham device was placed over P4 to stimulate the right PPC (Brodmann area 39) [27,29], and over C6 to stimulate the right STG (Brodmann areas 22 and 42) [36,37] according to the International 10–20 system for electrode placement, similar to previous rTMS and tDCS studies. tSMS effects are polarity independent [1] and, thus, we used only south polarity for all experiments. Sham stimulation was performed on P4 in eight participants and on C6 in another eight participants. The real and sham stimulations were performed in a random order. As reported in our previous study, the static magnetic field does not interfere with biological tissue at all; thus, the subjects cannot determine whether the object placed on the scalp is a magnet (real) or a non-magnetic material (sham) [1]. Since the visual stimulus presentation system created in this study provided the final score automatically, examiner bias was considered to be minimal.

### 2.4. Line Length Judgement Task

**Figure 1:**
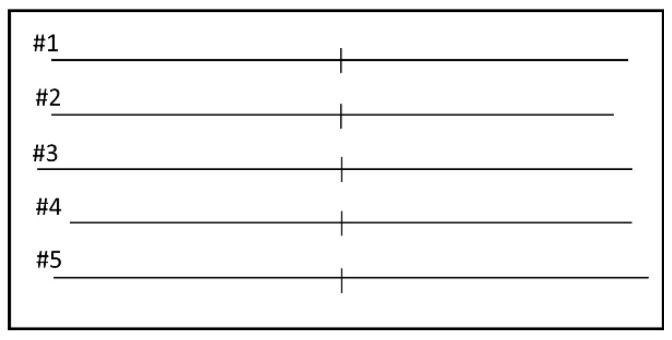
Line length judgement task. #1. Rt. and Lt. segments = 75 mm (Equally bisected). #2. Rt. = 70 mm; Lt. = 75 mm (Lt. longer). #3. Rt. = 75 mm; Lt. = 80 mm (Lt. longer). #4. Rt. = 75 mm; Lt. = 70 mm (Rt. longer). #5. Rt. = 80 mm; Lt. = 75 mm (Rt. longer).

The visual stimuli and their presentation method were based on a previous study [34]. 1 mm thick, 145 to 155 mm long horizontal lines transected by a 1 mm thick, 10 mm long vertical line was presented on the center of the monitor (12-inch FHD (1920 × 1080 dots), Refresh rate of 59 Hz). At each trial, one of five lines was presented, differing in the position of the vertical transector (at midpoint, rightward, or leftward) and in the overall length of the line and of its right and left segments (Figure 1).

To prevent eye scanning, the visual stimulus was presented for 150 ms (tachistoscopic presentation). Prior to the stimulus presentation, the subject was required to look at a fixation (an upward pointing arrow), which disappeared after appearance of the visual stimulus. After the stimulus presentation, the subject was required to make decision orally about the respective length of the two segments. There were three possible responses: equal, longer right, or longer left. In all task sessions, the subject performed 30 trials in a random order (10 trials with line #1 and five trials with lines #2–5). The interstimulus interval varied randomly between 8–12 s. The visual stimuli were presented using Psychopy 2.0.1 (Open Science Tools, Nottingham, UK) [38], an open source application for building a psychological experiment environment based on the Python language.

### 2.5. Data and Statical Analyses

#### 2.5.1. Scoring of Task Performance

The performance of the subject in each trial was scored as in Table 1. 

#### 2.5.2. Preliminary Experiment

A single measure of the interclass correlation coefficient, ICC (2,1), was used to measure the reproducibility of the task score (response) between two sessions for each stimulus presentation time. We examined the effect of stimulus presentation time (50 ms, 150 ms, and 300 ms) on the task score using a one-way repeated-measures analysis of variance (ANOVA). We also examine the effect of task session (1st to 6th) on the task score using a one-way repeated-measures ANOVA.

#### 2.5.3. Intervention Experiment

All data were expressed as the mean ± standard error of the mean (SEM). The normal distribution was confirmed using the Kolmogorov–Smirnov test. The effects of stimulation site (P4, C6, and Sham) and time (pre, post, and post 10) on the task score were analyzed using a two-way repeated-measures ANOVA. Post hoc differences were further analyzed with Bonferroni’s test. All analyses were performed with IBM SPSS Statistics software version 21 (SPSS; IBM, Armonk, NY, USA), and the significance level was set at 5%.

## 3. Results

### 3.1. Inter-Trial Reproducibility of the Score in Line Length Judgement Task

**Figure 2:**
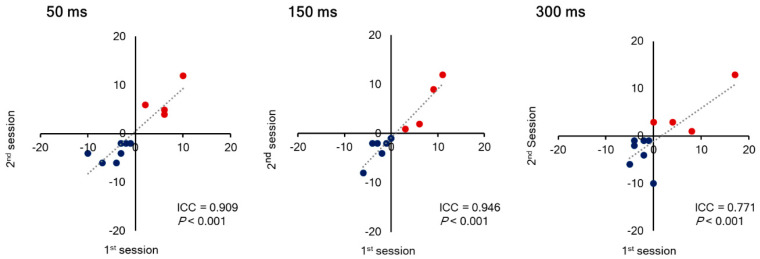
Relationship of line length judgement task score between 1st and 2nd preliminary sessions. The subjects plotted in the first quadrant show a rightward bias (red circles), and the subjects plotted in the third quadrant show a leftward bias (blue circles).

**Figure 3:**
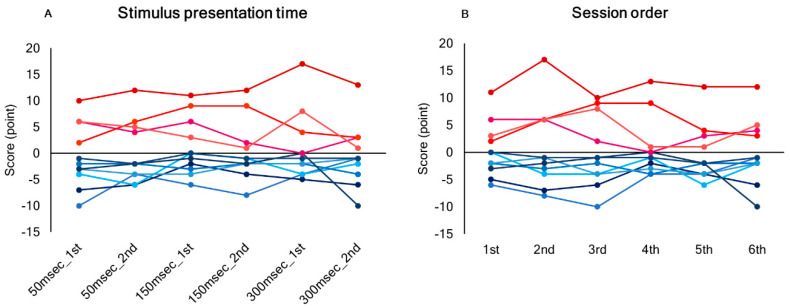
Individual scores of line length judgement task compared between (**A**) stimulus presentation times and (**B**) for session order (between 6 preliminary sessions). The red lines indicate a rightward bias, and the blue lines indicate a leftward bias.

Seven out of 11 subjects (4 males and 3 females) consistently underestimated the length of the right-side segment (judging lines to be exactly pre-bisected when the vertical transector was located to the left of the midpoint, or judging left-side segment to be longer when the vertical transector was located at the midpoint) (Leftward bias). In contrast, the remaining four subjects (2 males and 2 females) showed rightward bias. Inter-trial reproducibility of the score in a line length judgement task was ICC (2,1) = 0.909 for a stimulus presentation time of 50 ms, 0.946 for 150 ms, and 0.771 for 300 ms (*p* < 0.01) (Figure 2). The highest reproducibility was demonstrated with a stimulus presentation time of 150 ms. There was no significant effect of stimulus presentation time or session order on the score (Figure 3). The tendency of a leftward or rightward bias was consistent across a stimulus presentation times and sessions.

### 3.2. Effects of tSMS on the Scores of Line Length Judgement Task

**Figure 4:**
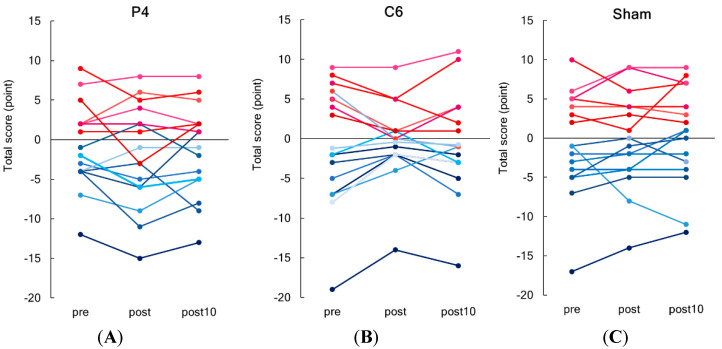
Serial changes in the individual total scores of line length judgement task before (pre), immediately after (post), and 10 min (post 10) after transcranial static magnetic stimulation (tSMS) for 20 min. Scatter plots present the individual values for each stimulation condition: (**A**) P4, (**B**) C6 and (**C**) sham stimulation. Nine subjects showed a rightward bias (reddish lines), and seven subjects showed a leftward bias (blueish lines).

Figure 4 shows serial changes in individual total scores of line length judgement task before (pre), immediately after (post), and 10 min (post 10) after tSMS/sham stimulation for a period of 20 min (tSMS over P4 and C6 and sham). In line with the preliminary experiment, we found two subgroups showing either a leftward (9 of 16, 6 males and 3 females) or rightward bias (7 of 16, 4 males and 3 females). The total score and the number of errors in total and in each score (−1, −2, +1, and +2) are summarized in Table 2 for the left-biased group and in Table 3 for the right-biased group.

**Figure 5:**
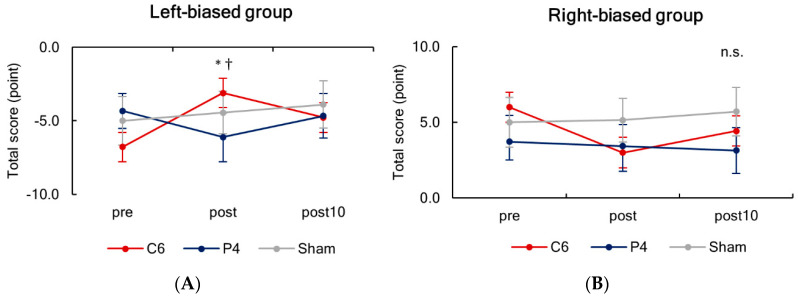
Serial changes in the total scores of line length judgement task before (pre), immediately after (post), and 10 min (post 10) after tSMS for 20 min, separately presented for the left-biased group (**A**) and right-biased group (**B**). In the left-biased group, the magnitude of bias was significantly reduced immediately after tSMS over C6, and the bias was significantly smaller for C6 as compared to P4 at post. * *p* < 0.001 vs. pre, ^†^ *p* < 0.05 vs. tSMS over P4.

**Figure A1:**
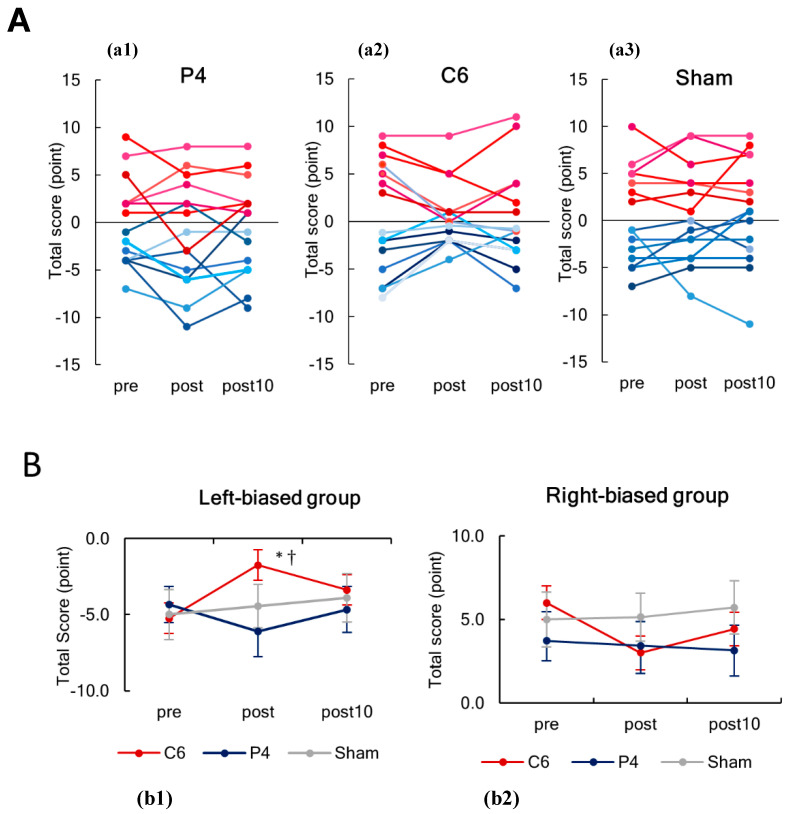
These figures show results without an outlier from the left-biased group. (**A**) Serial changes in the individual total scores of line length judgement task before (pre), immediately after (post), and 10 min (post 10) after tSMS for 20 min. Scatter plots present the individual values for each stimulation condition: (**a1**) P4, (**a2**) C6 and (**a3**) sham stimulation. Nine subjects showed a rightward bias (reddish lines), and seven subjects showed a leftward bias (blue lines). (**B**) Serial changes in the total scores of line length judgement task before (pre), immediately after (post), and 10 min (post 10) after tSMS for 20 min, separately presented for the left-biased group (**b1**) and right-biased group (**b2**). In the left-biased group, the magnitude of bias was significantly reduced immediately after tSMS over C6, and the bias was significantly smaller for C6 as compared to P4 at post. * *p* < 0.001 vs. pre, ^†^ *p* < 0.05 vs. tSMS over P4.

Since including the task scores from two subgroups in one analysis can result in the mean total score close to zero, we analyzed the effects of stimulation site and time separately for two subgroups. The two-way repeated-measures ANOVA revealed a significant interaction between stimulation site and time for the left-biased group (F_(4,32)_ = 2.898, *p* = 0.037, η^2^ = 0.266). For the tSMS over C6, a post hoc analysis showed a significant difference between before and immediately after 20 min of tSMS (post) (*p* < 0.001) (Figure 5). In addition, at immediately after 20 min of stimulation, a significant difference was revealed between two tSMS stimulation conditions (C6 and P4). No effect of stimulation site or time was observed for the right-biased group. Analysis without an outlier from the left-biased group can be found in Appendix A Figure A1.

## 4. Discussion

In this study, 9 of 16 subjects consistently underestimated the length of the right-side segment in line length judgement task, and the remaining 7 subjects consistently underestimated the length of the left-side segment. In these subjects showing the leftward bias, there was an improvement in the task score after an application of tSMS over C6 but not over P4 or after sham stimulation. In the other subjects showing the rightward bias, no significant intervention effect was observed with any stimulation.

### 4.1. Leftward and Rightward Biases in Healthy Individuals

Neurologically healthy individuals tend to show a leftward bias [39]. This phenomenon is known as pseudoneglect and can be observed in various visuo-spatial tasks [40,41]. In contrast to this previous observation, 7 of 16 subjects showed a rightward bias in the present study. Although at a first glance our finding seems contradictory, enormous individual differences have been observed in a relatively large number of previous studies on pseudoneglect [42,43,44,45]. In particular, Manning and colleagues found a rightward bias in 10 of 22 subjects with a large between-subject variability and proposed that this large variability could be the cause of frequent unsuccessful replication of leftward bias in healthy individuals [46]. Also, Jewell and McCourt pointed out in their review that, in addition to the problem of individual differences, scanning, sex, age, and hand dominance could influence the amplitude of pseudoneglect, even though their meta-analysis demonstrated the existence of leftward bias [41]. Thus, what we observed in the present study is not necessarily unique. Supportively, in our preliminary study, all the subjects consistently showed either a leftward or rightward bias over all sessions regardless of the stimulus presentation time. Furthermore, intersession reproducibility of the bias was quite high. From these data, there are assumed to be a certain number of individuals who show a rightward bias. Moreover, the high consistency and reproducibility suggest that the change in the task score by an application of tSMS over C6 in the left-biased group was not due to changes in attention, habituation, or fatigue resulting from repeated sessions.

### 4.2. Putative Mechanisms Underlying Change in the Score of Line Length Judgement Task by tSMS Over C6

SMFs have constant intensity and direction over time with a frequency of 0 Hz, and are different from electromagnetic fields that vary over time. Although growing evidence indicates that SMFs influence the central nervous system, it has not been fully understood exactly how this occurs. In a recent review on the effectiveness of SMFs at cellular level, SMFs were ensured to have an impact on cellular systems [47]. In particular, radial pair recombination and biomolecules reorientation by diamagnetic anisotropy effects were proposed to be conclusive as such SMFs’ effectiveness was consistently reported in all the previous studies: these changes subsequently result in susceptibility of biomolecules, intracellular structural modifications, and changes in the enzymatic reactions [47]. Rosen and colleagues also hypothesized that diamagnetic anisotropy by SMFs can cause a reorientation of phospholipids and hence an alteration of ion channels within them. Indeed, there is evidence that SMFs can influence membrane resting and action potentials by altering voltage-gated potassium [48], sodium [49], and calcium [50] channels. Another possibility is that the gradient of SMFs pushes calcium ions (positive ions) away from astrocytes, reducing the possibility of glutamate release [1]. Additionally, a recent study proposed a new hypothesis that magnetic pressure associated with gradients of the Zeeman influences a surface tension on the structure of the channel proteins, consequently altering the kinetics of voltage channel gating mechanics [51]. At current understanding, even though there are many hypotheses, there is no firm conclusion as to what mechanism is responsible for the change in the central nervous system by tSMS. Nevertheless, tSMS has been consistently demonstrated to have an inhibitory effect on the cortical excitability in various areas, including the motor [1,11], somatosensory [14,52], visual [53,54], and temporal [55] cortices, as well as SMA [16,17] and cerebellar region [56].

In the present study, tSMS applied over C6 (right STG) but not P4 (right PPC) altered the score of line length judgement task. Specifically, in the left-biased (right spatial neglect) group, the magnitude of bias or neglect was reduced immediately after tSMS over C6. Considering the inhibitory effect that tSMS has on the cortical excitability, it was expected that tSMS over the right PPC would reduce the leftward bias as in a previous study demonstrating improved leftward bias in healthy adults by inhibitory rTMS over the right PPC [34]. This contradictory result is hard to be explained, but one possibility is that tSMS was not strong enough to modulate the PPC. It is conceivable that a sufficient magnetic flux density did not reach into the PPC. Meanwhile, we find new evidence that inhibition of the right STG by tSMS can improve a leftward bias in healthy adults, which may be attributed to a relatively new hypothesis that USN is caused by lesions in the ventral attentional network, including the inferior parietal lobule, STG, and inferior frontal gyrus [57,58]. Following a finding that most post-stroke patients with USN had lesions in the ventral attentional network and not in dorsal attentional network consisting of the superior parietal lobule, frontal eye field, and intraparietal sulcus, Corbetta and colleagues hypothesized that lesions in the ventral attentional network bring about dysfunction of the dorsal parietal area, which consequently mediates a rightward bias in post-stroke patients [57,58]. Indeed, there is limited but supportive evidence of possible linkages between ventral and dorsal brain regions [59]. Therefore, we hypothesize that tSMS-induced reduction in the excitability of the STG, a part of the ventral attentional network, caused a change in the function of the dorsal parietal area, as a result inducing a rightward bias or, in other words, improving a leftward bias. Alternatively, tSMS over C6 might have influenced the superior longitudinal fasciculus that anatomically supports the ventral and dorsal attentional networks, as some research argues that lesions in the fasciculus is involved in the occurrence of USN [60]. However, in accordance with Coulomb’s law, the magnetic field strength decreases in inverse proportion to the square of the distance, and it is confirmed that the magnetic field strength of about 500 mT at the surface decreases to a low level (under 50 mT) at 5 cm from the magnet surface in actual measurement [13,61] and computer simulation [62,63,64]. Hence, we doubt that tSMS reached into the superior longitudinal fasciculus, a deep white matter tract. Additionally, the intervention effect of tSMS over C6 was transient, and there was no prolonged after-stimulation effect in the present study. As indicated in previous tSMS studies, when tSMS is applied over the motor cortex for less than 20 min, its after-stimulation effect lasts only for a few minutes. On the other hand, when the application time is over 30 min, the after effect can be as long as 30 min [12]. It is necessary to investigate whether cortical plastic change occurs only with long-term stimulation and also whether longer tSMS stimulation of the temporal cortex is necessary for a long-lasting change in visuo-spatial cognition.

### 4.3. Clinical Application

The incident of USN after a stroke in the right hemisphere ranges from 30% to 81% [65,66,67], and this disorder can be chronic (more than 1 year after stroke) in one third of the patients [68]. To alleviate the symptoms of neglect, various therapeutic interventions, such as visuospatial training, prism adaptation, and pharmacologic treatments, have been examined in research studies and also attempted in clinical practice [69]. However, no treatment for neglect has been established thus far. As reported in three recent reviews about the effectiveness of NIBS on USN symptoms [29,31,70], inhibitory rTMS is one of the promising tools for future treatment, but TMS is expensive and requires a quite high-level of technical skill for its use. On the other hand, the NdFeB magnet is an inexpensive industrial product that is easily available, and application of the magnet on the scalp does not require a high operational skill. Therefore, if tSMS applied over right hemisphere (either over C5 or P3) is found to attenuate neglect symptoms in post-stroke patients in future studies, then this new NIBS technique may become a clinically useful tool. Furthermore, it may be used as an in-home intervention along with other rehabilitation exercise programs. Meanwhile, as shown in this and previous tSMS studies, a short after-effect may be a disadvantage compared with a traditional NIBS tools. Since tSMS over the motor cortex for 30 min has been demonstrated to result in an after-effect of 30 min [10], it should be investigated whether a longer duration of tSMS over the temporal cortex similarly induces long-lasting after-effects on visuo-spatial cognitive function. Given the short after-effect, another potential clinical use could be to repeat tSMS in combination with rehabilitation for 4–8 weeks, like interventions using rTMS and tDCS [71,72]. Clinical investigation is clearly warranted to confirm the potential benefit of using tSMS.

## 5. Conclusions

The present study demonstrated that application of tSMS over the right STG reduces a leftward bias and thus modulates visuo-spatial cognition in healthy adults. Further study is needed to clarify the neurophysiological mechanism underlying this modulation by tSMS over the temporal but not parietal cortex.

## Figures and Tables

**Table 1:** Score and response in line length judgement task.

Score	Response
0	Correct response
+1	Right segment of line #1 judged longer, or left and right segments of lines #2 and #3 judged equal (rightward bias)
−1	Left segment of line #1 judged longer, or left and right segments of lines #4 and #5 judged equal (leftward bias)
+2	Right segment of lines #2 and #3 judged longer (rightward bias)
−2	Left segment of lines #4 and #5 judged longer (leftward bias)

Line number see caption of Figure 1.

**Table 2:** The total score and the number of errors in the line length judgement task for the left-biased group (mean ± standard error of the mean (SEM)). *n* = 9. * *p* < 0.001 vs. pre, ^†^ *p* < 0.05 vs. tSMS over P4.

Stim. Site	P4			C6			Sham		
Time	Pre	Post	Post 10	Pre	Post	Post 10	Pre	Post	Post 10
Total Score	−4.3 ± 1.2	−6.1 ± 1.7	−4.7 ± 1.5	−6.8 ± 1.7	−3.1 ± 1.4 *^†^	−4.8 ± 1.5	−5.0 ± 1.6	−4.4 ± 1.4	−3.9 ± 1.6
Total error	8.3 ± 1.2	8.9 ± 1.4	7.7 ± 1.4	8.7 ± 1.5	5.4 ± 1.2	8.1 ± 1.2	7.6 ± 1.2	6.0 ± 1.5	6.0 ± 1.1
−1 Error	8.3 ± 0.8	8.9 ± 1.3	7.7 ± 1.3	7.4 ± 1.4	5.4 ± 1.1	8.1 ± 1.1	7.6 ± 1.2	6.0 ± 1.1	6.0 ± 1.1
−2 Error	0.1 ± 0.1	0.2 ± 0.1	0.0 ± 0.10	0.1 ± 0.1	0.1 ± 0.1	0.2 ± 0.1	0.1 ± 0.1	0.2 ± 0.1	0.1 ± 0.1
+1 Error	2.2 ± 0.9	1.4 ± 0.8	1.4 ± 0.8	0.8 ± 0.4	1.0 ± 0.4	1.2 ± 0.5	1.2 ± 0.5	0.9 ± 0.5	1.7 ± 0.5
+2 Error	0.0 ± 0.0	0.0 ± 0.0	0.0 ± 0.0	0.1 ± 0.1	0.0 ± 0.0	0.0 ± 0.0	0.0 ± 0.0	0.0 ± 0.0	0.0 ± 0.0

**Table 3:** The total score and the number of errors in the line length judgement task for the right-biased group (mean ± SEM). *n* = 7.

Stim. Site	P4			C6			Sham		
Time	Pre	Post	Post 10	Pre	Post	Post 10	Pre	Post	Post 10
Total Score	3.7 ± 1.3	3.4 ± 1.3	3.1 ± 1.3	6.0 ± 0.8	3.0 ± 1.3	4.4 ± 1.7	5.0 ± 1.0	5.1 ± 1.1	5.7 ± 1.0
Total error	5.9 ± 0.9	6.1 ± 1.5	6.9 ± 1.4	7.9 ± 1.3	7.9 ± 1.8	6.9 ± 1.5	7.6 ± 1.4	6.0 ± 1.0	6.0 ± 1.1
−1 Error	1.0 ± 0.6	1.4 ± 0.7	1.9 ± 0.8	0.9 ± 0.3	2.4 ± 0.6	1.4 ± 0.4	1.1 ± 0.5	0.9 ± 0.4	1.0 ± 0.3
−2 Error	0.0 ± 0.0	0.0 ± 0.0	0.0 ± 0.0	0.0 ± 0.0	0.0 ± 0.0	0.0 ± 0.0	0.0 ± 0.0	0.0 ± 0.0	0.0 ± 0.0
+1 Error	4.6 ± 0.9	4.6 ± 1.3	4.4 ± 1.3	6.6 ± 1.0	5.4 ± 1.5	5.9 ± 1.6	5.9 ± 1.0	6.0 ± 1.0	6.7 ± 1.0
+2 Error	0.0 ± 0.0	0.1 ± 0.1	0.0 ± 0.0	0.1 ± 0.1	0.0 ± 0.0	0.0 ± 0.0	0.1 ± 0.1	0.0 ± 0.0	0.0 ± 0.0
